# Leveraging collateral sensitivity to counteract the evolution of bacteriophage resistance in bacteria

**DOI:** 10.1002/mlf2.70003

**Published:** 2025-03-18

**Authors:** Yongqi Mu, Yuqin Song, Xueru Tian, Zixuan Ding, Shigang Yao, Yi Li, Chao Wang, Dawei Wei, Waldemar Vollmer, Gang Zhang, Jie Feng

**Affiliations:** ^1^ State Key Laboratory of Microbial Resources, Institute of Microbiology Chinese Academy of Sciences Beijing China; ^2^ College of Life Science University of Chinese Academy of Sciences Beijing China; ^3^ School of Clinical and Basic Medical Sciences Shandong First Medical University & Shandong Academy of Medical Sciences Jinan China; ^4^ Institute for Molecular Bioscience The University of Queensland Brisbane Queensland Australia; ^5^ Centre for Bacterial Cell Biology, Biosciences Institute Newcastle University Newcastle upon Tyne UK

**Keywords:** antibiotic resistance, bacteriophage (phage) cocktails, collateral sensitivity, evolution, *Klebsiella pneumoniae*

## Abstract

The escalating antibiotic resistance crisis poses a major global health threat. Bacteriophage therapy offers a promising alternative for combating multidrug‐resistant infections. However, bacterial resistance to phages remains a significant hurdle. Innovative strategies are needed to overcome this challenge. In this study, we developed a phage cocktail based on our phage library, consisting of three phages that suppressed phage resistance of carbapenem‐resistant hypervirulent *Klebsiella pneumoniae* (CR‐hvKp). This cocktail capitalized on dual instances of collateral sensitivity, thereby constraining the evolution of phage resistance. The first‐layered collateral sensitivity arose from overlapping coverage between capsular polysaccharide (CPS) and lipopolysaccharide (LPS), rendering the bacteria resistant to CPS‐binding phages but more susceptible to LPS‐binding phages. The second‐layered collateral sensitivity resulted from an O serotype switch (from O1 to O2), causing resistance to O1 antigen‐binding phages but increasing susceptibility to phages that target the O2 antigen. This dual‐layered collateral sensitivity phage cocktail effectively mitigated infection caused by CR‐hvKp in mice. Our research highlights the importance of the collateral sensitivity mechanism in counteracting the evolution of phage resistance and offers a sophisticated strategy for configuring phage cocktails to eliminate bacterial resistance.

## Impact statement

Phage therapy offers a promising solution for tackling multidrug‐resistant infections, but bacterial resistance to phages remains a significant obstacle. In this study, we harnessed collateral sensitivity to design a phage cocktail that effectively prevented phage resistance in carbapenem‐resistant *Klebsiella pneumoniae*. Building on this groundwork, we propose a novel approach for creating a phage cocktail by exploiting collateral sensitivity, providing a flexible and scalable strategy to overcome clinical challenges in phage therapy and ultimately enhancing its effectiveness against drug‐resistant bacterial infections.

## INTRODUCTION

The growing crisis of antibiotic resistance presents a substantial risk to worldwide public health, highlighting the critical demand for therapeutic alternatives. Recently, phage therapy has gained attention as a promising alternative^1^. Bacteriophages (phages) are viruses that prey on bacteria and offer a unique mechanism of action that is distinct from antibiotics, enabling them to eliminate bacteria without being hindered by existing antibiotic resistance^2^, ^3^. However, bacteria can easily develop resistance to phages. In a variety of systems, the occurrence of phage resistance is quite common, with frequencies ranging from 10^−5^ to 10^−7^, akin to antibiotic resistance, though there is significant variation^4^. Mutations leading to changes on the cell surface that prevent phage attachment represent the most straightforward mechanisms through which bacteria can develop resistance to phages. The receptors for phages on bacterial surfaces mainly include lipopolysaccharide (LPS), capsular polysaccharide (CPS), or outer membrane protein (porin)^5^, ^6^. The variety of phage receptors on bacterial surfaces creates the opportunity to formulate phage cocktails comprising several phages, with each one aiming at a distinct receptor present on the bacterial strain. Implementing this tactic could significantly lower the chances of the emergence of phage‐resistant bacterial mutants^7^, ^8^. Nonetheless, there is a need to improve the effectiveness of these phage cocktails and to overcome resistance challenges.

Numerous studies have systematically investigated drug combinations, uncovering a significant phenomenon known as collateral sensitivity. This occurs when pathogens like *Pseudomonas aeruginosa* and *Escherichia coli*, upon developing resistance to certain antibiotics, inadvertently become more susceptible to others^9^, ^10^, which promotes crafting drug combinations in a more rational and effective manner. In a recent study, vancomycin‐resistant *Enterococcus faecium* (VRE) displayed increased susceptibility to pleuromutilin, which proved more effective than linezolid‐based therapy in clearing VRE colonization and septicemia in mouse models^11^. Furthermore, the principle of collateral sensitivity has been identified in cancer cell lines^12^ and proven effective in an animal model for treating Philadelphia chromosome (Ph)+ acute lymphoblastic leukemia with tyrosine kinase inhibitor drugs^13^. Building on this concept, we hypothesized that collateral sensitivity could be a tactic when designing phage combination therapies to combat phage resistance.

*Klebsiella pneumoniae* (Kp) is a significant clinical pathogen that plays a role in both healthcare‐associated and community‐acquired infections. The hypervirulent strain of *K. pneumoniae* (hvKp) raised particular concerns due to its link with severe community‐acquired infections, especially hepatic abscesses^14^. The hvKp isolates frequently exhibit two K serotypes, K1 and K2, and three O serotypes, O1, O2, and O3^15^. They also often carry virulence genes located on plasmids, together with K1 and K2 CPS to contribute to the hypervirulent phenotype^14^. While the initial hvKp isolates were sensitive to antimicrobials, recent studies showed the frequent emergence of carbapenem‐resistant hypervirulent *K. pneumoniae* (CR‐hvKp)^16^, ^17^, presenting greater challenges for clinical treatment. On the other hand, the human gut could act as a reservoir for hvKp, with initial colonization upon hospital admission significantly associated with subsequent infections^18^, ^19^, ^20^. Employing phage therapy to eliminate pathogens like hvKp in the gut could be advantageous in preserving the natural microbial community and reducing the likelihood of widespread resistance^21^, ^22^. However, the full implementation of phage therapy encountered significant obstacles, including the identification of effective phage candidates for particular bacterial strains and the challenge of phage resistance development.

To tackle the issue of phage resistance in CR‐hvKp, we developed a phage cocktail based on our comprehensive phage library, consisting of three unique phages that suppressed phage resistance. This cocktail capitalized on dual instances of collateral sensitivity, thereby constraining the development of phage resistance in CR‐hvKp. The loss of CPS led to resistance to CPS‐binding phages but increased susceptibility to non‐CPS binding phages, indicative of the first‐layered collateral sensitivity. The O‐serotype switch (O1 to O2) was responsible for the second‐layered collateral sensitivity. This dual‐layered collateral sensitivity phage cocktail effectively mitigated infection caused by CR‐hvKp in mice. Collectively, our research provides fresh perspectives on employing evolutionary trade‐offs to inhibit phage resistance.

## RESULTS

### Screening phages for a cocktail against hvKp

**Figure 1:**
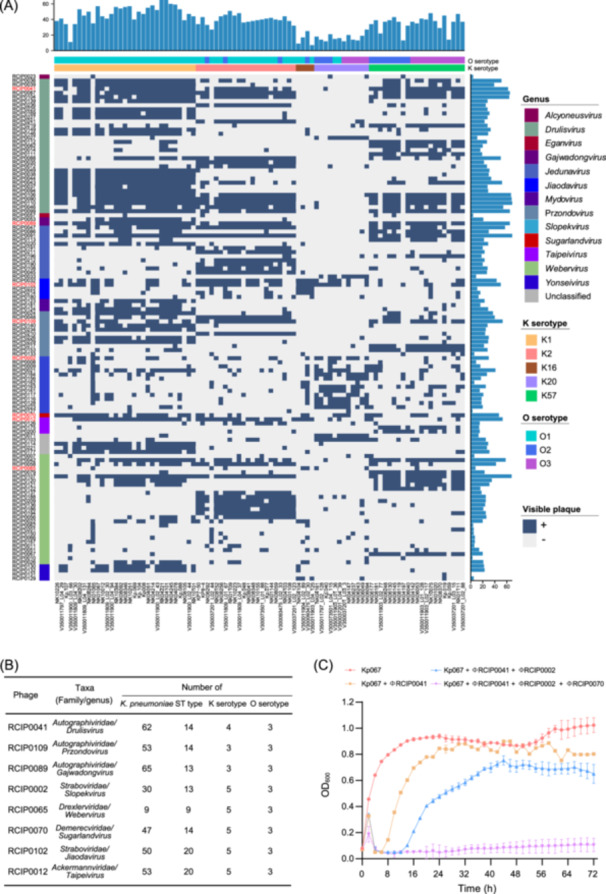
Screening phages for a cocktail against hvKp. (A) The host–phage incidence matrix. Each row corresponds to a specific phage and each column corresponds to a different hvKp strain. The presence of blue‐colored squares within the matrix highlights the visible plaques formed as a result of interactions between hosts and phages. Atop the matrix, a bar chart details the count of phages capable of infecting each hvKp strain, while a bar chart to the right of the matrix displays the number of hvKp strains that could be lysed by a single phage. Color‐coded strips situated above the matrix serve to categorize the *Klebsiella pneumoniae* strains by their K serotypes and O serotypes, and color‐coded strips on the left side of the matrix are employed to denote the genera of the phages. (B) Detailed information on the eight phages selected as potential components for the cocktail formulation, including their diverse taxa and ability to lyse *K. pneumoniae* strains. (C) Growth curves for Kp067 cultures, including noninfected samples and those infected with a single phage or multiple phage combinations, over 72 h. The *y*‐axis represents the optical density (OD_600_). The displayed data represent the means ± standard deviations (SD) from three independent experiments.

To develop a phage cocktail capable of targeting a broad range of hvKp strains and suppressing the emergence of phage‐resistant mutants, we screened a curated library of 124 lytic phages encompassing nine families and thirteen genera, which represent a significant portion of the known *Klebsiella*‐infecting phage diversity (Figure S1A). We assessed the host range and lytic potency of these phages using spot tests against a panel of 90 hvKp strains, comprising 10 gut‐derived and 80 clinical isolates (Table S1). These strains, which harbor virulence‐associated biomarkers such as *rmpA*/*rmpA2*, *iuc*, and/or *iro* loci^14^, ^18^, were classified into K serotypes (K1, K2, K57, K16, and K20) and O serotypes (O1, O2, and O3). They encompassed 21 distinct sequence types (STs), with ST23, ST218, and ST412 being predominant. Of note, 24 of the 90 strains (27%) possessed genes conferring resistance to carbapenems (*bla*_KPC_, *bla*_NDM_, *bla*_IMP_, and *bla*_OXA_) or colistin (*mcr*), underscoring the threat posed by these CR‐hvKp isolates (Figure S1B). Our comprehensive phage–host interaction matrix revealed 3366 lytic phenotypes out of 11,160 potential interactions (Figure 1A). We found that each hvKp strain was susceptible to being lysed by a minimum of 6 distinct phages, and each phage exhibited lytic activity against a range of 1 to 69 strains. Considering the phage phylogeny and breadth of the host range, we selected 8 phage candidates for the cocktail aimed at eradicating hvKp (Figures 1B and S2). These phages, belonging to 5 families and 8 genera, demonstrated the capacity to lyse 9 to 65 of the tested strains (Figure 1A, Table S2), covering the most prevalent STs and serotypes.

### Phage combination suppresses phage resistance of CR‐hvKp

We selected *K. pneumoniae* NK01067 (abbreviated as Kp067) as the experimental strain, which carries the *bla*_NDM‐1_ gene responsible for carbapenem resistance, and a virulence plasmid, which contributes to hypermucoviscosity. Strain Kp067 was classified as K1 and O1 serotypes. We evaluated the efficacy of 18 unique phage combinations against Kp067. As shown in the bacterial growth curves (Figure S3), all phage combinations delayed the onset of bacterial regrowth compared to single‐phage applications. Furthermore, we found that one combination of three phages completely inhibited the growth of Kp067 within 24 h, with no significant resurgence observed even after an extended incubation period of 72 h (Figure 1C). This combination consists of three phages, ΦRCIP0041, ΦRCIP0002, and ΦRCIP0070, which belong to the *Drulisvirus*, *Slopekvirus*, and *Sugarlandvirus* genera, respectively (Figure 1B). Morphological observation using transmission electron microscopy (TEM) demonstrated that ΦRCIP0041, ΦRCIP0002, and ΦRCIP0070 are *Podoviridae*, *Myoviridae*, and *Siphoviridae* phage, respectively (Figure S2B).

### Collateral sensitivity constraints phage resistance evolution of the CR‐hvKp

**Figure 2:**
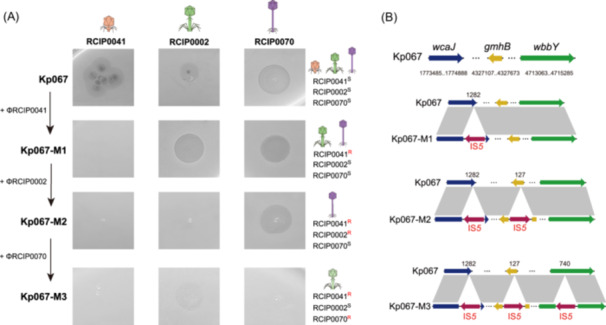
Evolution of Kp067 to generate phage resistance. (A) Evolutionary trajectory of Kp067 examined under sequential exposure to each of the three phages. Visible plaque represents phage sensitivity, while no plaque represents phage resistance. The orange‐colored phages represent the *Podoviruses* phage ΦRCIP0041, while the green‐colored ones represent the *Myoviridae* phage ΦRCIP0002, and the purple‐colored ones represent the *Siphoviridae* phage ΦRCIP0070. R, resistance to phage; S, sensitivity to phage. (B) Schematic representation of IS*5* insertion sites in Kp067 phage‐resistant mutants. The arrows in colors of blue, tawny, and green, respectively, represent the *wcaJ*, *gmhB*, and *wbbY*, the corresponding genes inserted by IS*5*, while the red arrows represent IS*5*. The numbers under the genes represent the coding regions on the chromosome of Kp067 for these three genes: *wcaJ*, 1773485..1774888; *gmhB*, 4327107..4327673; and *wbbY*, 4713063..4715285. The numbers above these genes donate the insertion site of the IS*5* element for each gene, for example, 1282; the IS*5* inserted into the 1282th bp of the *wcaJ* gene.

To elucidate the mechanisms underlying the prevention of resistance development in the CR‐hvKp by the phage cocktail, we performed sequential evolutionary experiments with Kp067 under the selective pressure of three different phages (Figures 2A and S4). Initially, Kp067 was co‐cultured with phage ΦRCIP0041, yielding the resistant mutant Kp067‐M1. Interestingly, ΦRCIP0002 and ΦRCIP0070 produced more distinct plaques on Kp067‐M1 compared to Kp067. Subsequent exposure of Kp067‐M1 to ΦRCIP0002 led to the screening of the resistant mutant Kp067‐M2, which was still vulnerable to being lysed by ΦRCIP0070. The final resistant mutant, Kp067‐M3, emerged upon challenging Kp067‐M2 with ΦRCIP0070. Interestingly, Kp067‐M3 exhibited resistance to ΦRCIP0070 but regained susceptibility to ΦRCIP0002.

To accurately measure the infection capabilities of the phages against the bacterial mutants, ΦRCIP0002 and ΦRCIP0070 phage lysates were subjected to 10‐fold serial dilutions and then plated together with Kp067 or its mutant Kp067‐M1 cultures. The results showed that phages ΦRCIP0002 and ΦRCIP0070 demonstrated an estimated 10^3^‐fold and 10^2^‐fold enhancement in plaque‐forming efficiency on the Kp067‐M1 cells compared to the Kp067 cells (Figure S5A). Similar experiments were conducted to assess the impact of ΦRCIP0070 and ΦRCIP0002 on the mutant strains Kp067‐M2 and Kp067‐M3 (Figure S5B). The phage ΦRCIP0070 was capable of producing progeny in the mutant strain Kp067‐M2, yet it failed to do so in Kp067‐M3. Conversely, ΦRCIP0002 successfully generated progeny in Kp067‐M3, but not in Kp067‐M2. Additionally, the results of the growth curve indicated that ΦRCIP0070 markedly suppressed the growth of Kp067‐M2, yet it had no discernible effect on Kp067‐M3. In contrast, ΦRCIP0002 substantially hindered the growth of Kp067‐M3 without affecting Kp067‐M2 (Figure S5C,D). This pattern of resistance evolution, where becoming resistant to one phage increases susceptibility to another, is referred to as collateral sensitivity.

### Genome analysis uncovers potential phage receptors in the CR‐hvKp

To elucidate the genetic basis of collateral sensitivity in phage‐resistant mutants of Kp067, bioinformatics analyses based on complete genomes of Kp067 and its derivative mutants were performed. An IS*5* transposable element was revealed inserting into specific genes across different mutants: the *wcaJ* gene in the mutant Kp067‐M1; both *wcaJ* and *gmhB* in Kp067‐M2; and *wcaJ*, *gmhB*, and *wbbY* in Kp067‐M3 (Figure 2B).

The *wcaJ* gene encodes undecaprenyl‐phosphate glucose‐1‐phosphate transferase^23^, ^24^, a key enzyme in the synthesis of CPS, suggesting that CPS components may act as the binding receptor for the phage ΦRCIP0041. The *gmhB* gene encodes the enzyme d,d‐heptose 1,7‐bisphosphate phosphatase, crucial for converting d,d‐heptose 1,7‐bisphosphate into d,d‐heptose 1‐phosphate^25^, ^26^, ^27^. This conversion is a significant step in the biosynthesis of the LPS core polysaccharide, indicating that the LPS core polysaccharide may serve as the receptor for the phage ΦRCIP0002. Furthermore, the mutant Kp067‐M3 contained an IS*5* insertion in the *wbbY* gene. The *wbbY* gene encodes a galactosyltransferase involved in the production of the LPS O1‐antigen surface polysaccharide^28^. This disruption suggested that the receptor for the phage ΦRCIP0070 may be located on the LPS O1‐antigen.

### Genetic landscape of phage interaction with CR‐hvKp mutants reveals collateral sensitivity pathways

**Figure 3:**
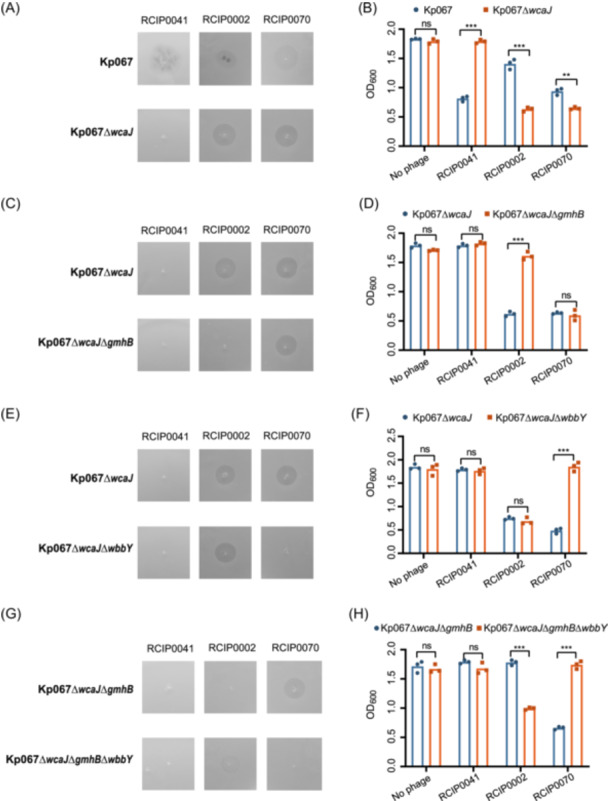
Pivotal role of genes *wcaJ*, *gmhB,* and *wbbY* in phage–host recognition and the collateral sensitivity. (A, C, E, and G) The lytic ability of the three phages to Kp067 and its gene knockout mutants on the solid medium measured by plaque assays. Visible plaque represents phage sensitivity, while no plaque represents phage resistance. (B, D, F, and H) Growth curves displaying the inhibition effect of the three phages against Kp067 and its gene knockout mutants in the liquid medium, corresponding to the plaque assays on the left panels. Data represent the means of three independent experiments. ns, no significant difference; **p < 0.01; ***p < 0.001.

To investigate the distinct roles of *wcaJ*, *gmhB*, and *wbbY* genes on phage resistance, we established a series of gene knockout strains: Kp067∆*wcaJ*, Kp067∆*wcaJ*∆*gmhB*, Kp067∆*wcaJ*∆*wbbY*, and Kp067∆*wcaJ*∆*gmhB*∆*wbbY*, deficient of one or more of the specified genes. These mutants exhibited lysis or lack of lysis phenotypes similar to those in evolutionary experiments, confirming that disrupting genes of *wcaJ*, *gmhB*, and/or *wbbY* was enough to elicit the observed phage resistance phenotypes (Figure 3). Deletion of *wcaJ* resulted in complete resistance to the phage ΦRCIP0041 (Figures 3A,B and S6), and introducing *wcaJ* into Kp067∆*wcaJ* restored susceptibility to the phage ΦRCIP0041 (Figure S7A), consistent with CPS being the receptor for this phage. Interestingly, phages ΦRCIP0002 and ΦRCIP0070 showed an increased lysis capacity in Kp067∆*wcaJ* cells compared to the Kp067 cells (Figure 3A,B). The CPS might obscure the partial receptors for ΦRCIP0002 and ΦRCIP0070, and the loss of CPS resulted in increased vulnerability to the two phages, thereby revealing the first‐layered collateral sensitivity.

GmhB was a partially redundant enzyme in the synthesis of ADP‐heptose for the LPS core polysaccharide, and deletion of *gmhB* gene could decrease the core‐oligosaccharide synthesis, leading to the formation of an altered LPS core^25^, ^26^, which was verified by LPS silver staining (Figure S8). The deletion of the *gmhB* gene led to resistance against the phage ΦRCIP0002 (Figures 3C,D and S6), and introducing *gmhB* into Kp067∆*wcaJ*∆*gmhB* restored susceptibility to the phage ΦRCIP0002 (Figure S7B), indicating that the phage targeted the LPS core oligosaccharide.

The O‐antigen polysaccharide (OPS) is linked to the lipid A‐core oligosaccharide component of LPS^29^, ^30^. The O2 serotype consists of only the O2 antigen, while the O1 antigen is covalently linked to the nonreducing end of the O2 antigen by WbbY, forming the O1 serotype^28^. The LPS silver staining revealed that the O antigen changed from high molecular weight to low molecular weight (corresponding to the O serotype switch from O1 to O2) when the *wbbY* gene was deleted (Figure S8). Inactivation of the *wbbY* gene in the Kp067∆*wcaJ* strain resulted in resistance to the phage ΦRCIP0070 (Figures 3E,F and S6). Conversely, reintroducing *wbbY* into this genetically modified strain restored its susceptibility to ΦRCIP0070 (Figure S7C), indicating that ΦRCIP0070 specifically targeted the O1 antigen. Consistent with the evolutionary screening experiment and genomic analysis, the deletion of *wbbY* in Kp067∆*wcaJ*∆*gmhB* reinstated sensitivity to ΦRCIP0002, demonstrating the second‐layered collateral sensitivity (Figures 3G,H and S6). Moreover, reintroducing *wbbY* into the Kp067∆*wcaJ*∆*gmhB*∆*wbbY* strain led to resistance against ΦRCIP0002 (Figure S7D). The absence of the O1 antigen exposed the underlying O2 antigen, suggesting that ΦRCIP0002 may target the O2 antigen. This series of genetic experiments elucidated the complex interplay between bacterial surface antigens and phages, uncovering the genetic underpinnings of collateral sensitivity mechanisms.

### Phage cocktail exhibiting collateral sensitivity effectively mitigates infection caused by the CR‐hvKp in mice

**Figure 4:**
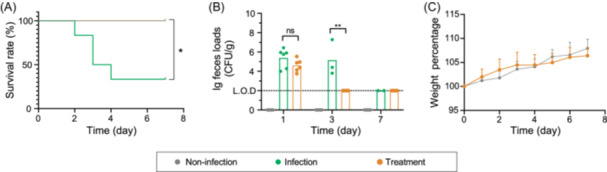
Phage cocktail with a collateral sensitivity to eradicate Kp067 in the mouse gut. (A) Survival rate of murine infection models of Kp067 with or without phage cocktail treatment within 7 days. Male BALB/c mice 6–8 weeks old were used for establishing murine models with 6 mice per group. The inoculation dose was 10^7^ CFU Kp067 cells. (B) Abundance of Kp067 in feces of experimental mice with or without the treatment of phage cocktail within 7 days. (C) Relative daily weight of the non‐infected mice and Kp067 infected mice with the phage cocktail treatment within 7 days. ns, no significant difference; *p < 0.05; **p < 0.01. Limit of detection (L.O.D) is 100 CFU g^−1^ feces.

Based on the dual‐layered collateral sensitivity mechanism, the phage cocktail, consisting of ΦRCIP0041, ΦRCIP0002, and ΦRCIP0070, could effectively limit the emergence of phage resistance, showing considerable promise for practical applications. To assess the therapeutic potential of our phage cocktail against infection caused by the CR‐hvKp (Kp067), specific pathogen‐free (SPF) BALB/c mice were first orally inoculated with Kp067 (1 × 10^7^ CFU). 1 h after inoculation, these mice were orally administered 1 × 10^7^ PFU of the phage cocktail. Within a 7‐day period, the CR‐hvKp strain Kp067 led to a high mortality rate of 67% for the mice in the infected group. In contrast, no mortalities were observed in either the phage treatment group or the non‐infected group (Figure 4A). Furthermore, the measurement for the abundance of Kp067 in fecal samples showed that from the first day, the phage cocktail could significantly reduce the Kp067 abundance in the treatment group. By the third day, Kp067 was undetectable in the fecal samples of the treatment group, significantly lower than those of the infected group (about 10^5^ CFU/g) (Figure 4B). Notably, there was a minimal alteration in the body weight percentages of mice between the phage treatment group and non‐infected group (Figure 4C). These results underscored the phage cocktail's efficacy in significantly reducing bacterial load and mitigating infection in mice, thereby demonstrating its potential as a therapeutic intervention against CR‐hvKp infections.

### Phage cocktail with collateral sensitivity counteracts the evolution of phage resistance in another hypervirulent *K. pneumoniae* strain

**Figure 5:**
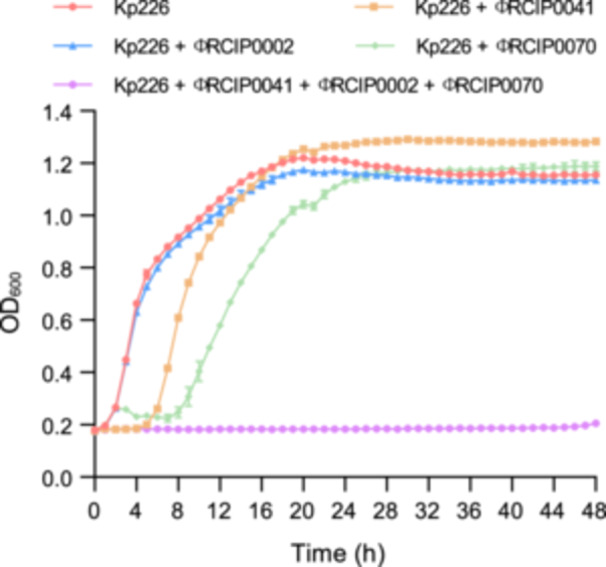
Phage cocktail with collateral sensitivity counteracts the evolution of phage resistance in *K. pneumoniae* strain Kp226. Growth curves of untreated or phage‐treated Kp226 cultures are shown. The OD_600_ of the *K. pneumoniae* strain Kp226 treated with different phages, and the phage cocktail was detected over 48 h. The displayed data represent the means ± standard deviations (SD) from three independent experiments.

The phage cocktail with collateral sensitivity could counteract the evolution of phage resistance in the Kp067 strain, which represents those with K1 and O1 serotypes. To explore the robust lytic ability of this “collateral sensitivity” cocktail to K1 and O1 serotype strains, we tested this cocktail against another clinical strain NK10226 (abbreviated as Kp226, K1, O1). As indicated by the growth curve, the phage cocktail with the three‐phage combination completely inhibited the growth of the strain Kp226 within 48 h, surpassing the results achieved with single‐phage treatments (Figure 5). Additionally, an analysis of 1760 complete *K. pneumoniae* genomes from the National Center for Biotechnology Information (NCBI) RefSeq database revealed that 31.8% (*n* = 560) of the strains harbored the O1 antigen (Table S3). These findings suggest that using a cocktail with collateral sensitivity could help limit the emergence of phage resistance in O1‐antigen strains.

## DISCUSSION

Antibiotic resistance sparked a renewed focus on employing phages as antimicrobial agents. However, the rapid phage resistance development still severely restricts the efficacy of phage treatments. Therefore, there is a pressing need to intensify efforts to address the challenges posed by phage resistance.

**Figure 6:**
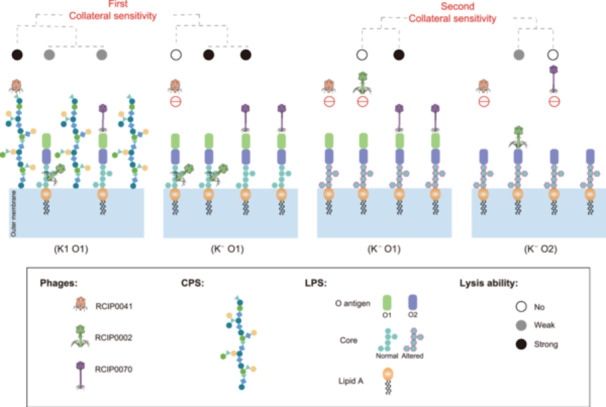
Schematic for the collateral sensitivity mechanism of the phage cocktail infecting *K. pneumoniae*. The CPS loss (K^−^) mediates the first collateral sensitivity, while the O1 to O2 serotype switch mediates the second collateral sensitivity. In addition, the phage RCIP0002 might have two binding sites, the LPS core oligosaccharide during the phase of first collateral sensitivity and the O2 antigen during the phase of second collateral sensitivity. CPS, capsular polysaccharide; LPS, lipopolysaccharide.

Collateral sensitivity represents an evolutionary trade‐off in which the development of resistance to one drug increases susceptibility to another. This concept has been leveraged in studies to mitigate drug resistance evolution in both pathogens^31^ and cancer cells^12^. These laboratory studies suggest that collateral sensitivity may be used in future therapeutic strategies, resulting in more effective utilization of existing anticancer medications and antibiotics^32^, ^33^. Phages rely on bacterial host receptors for recognition and adsorption during attachment. Consequently, mutations in these receptor genes serve as a primary mechanism of bacterial resistance to phages^5^. To counteract the phage resistance, phage cocktails harboring multiple phages were used^3^, ^4^. However, simply increasing the phage number cannot easily eliminate the resistance^3^, ^4^. The different receptor composition and their relationship required consideration. This study demonstrated that the loss of CPS led to resistance against CPS‐binding phages but increased the vulnerability to LPS‐binding phages, showcasing the first‐layered collateral sensitivity. Similarly, the loss of the O1 antigen conferred resistance to O1 antigen‐binding phages but introduced susceptibility to O2 antigen‐binding phages, revealing the second‐layered collateral sensitivity (Figure 6). This was supported by two recent studies. One study revealed that resistance developed through receptor modification against capsule‐targeting phages not only introduced cross‐resistance to phages that target the same receptor but also increased sensitivity to phages that utilize different receptors^34^. The other study on the development of phage resistance in *Salmonella typhimurium* showed that employing phage combinations that exploited collateral sensitivity could significantly reduce the emergence of resistance^35^. Therefore, we propose to adopt the collateral sensitivity strategy for phage selection to combat pathogens.

Bacterial cell‐surface polysaccharides, mainly encompassing K‐antigen (CPS) and O‐antigen (LPS) polysaccharides, are important for the pathogen's survival during infection^36^. They also served as the primary receptors for phages that preyed on *K. pneumoniae* or *E. coli* ^37^, ^38^, ^39^. Our study revealed that phages with the ability to target LPS displayed a markedly improved capacity to lyse CPS‐deficient mutants. A potential rational explanation is the increased exposure of LPS following the CPS loss. Employing a combination of phages that collectively target both LPS and CPS could serve as an effective tactic for developing a *K. pneumoniae* phage cocktail designed to alleviate resistance. More importantly, our findings extend the insights of collateral sensitivity involving the O1 serotype strains of *K. pneumoniae*. The absence of the O1 antigen, which resulted in the O serotype switch of O1 to O2, conferred resistance to phages binding to the O1 antigen while simultaneously increasing vulnerability to phages that targeted the O2 antigen. Our results highlight the potential of exploiting collateral sensitivity to boost the efficacy of phage therapy against specific bacterial targets, offering a sophisticated strategy for tackling bacterial resistance to phages.

## MATERIALS AND METHODS

### Bacterial strains and culture conditions

A collection of 90 hvKp strains was selected, consisting of 10 gut‐derived and 80 clinical isolates. The 10 gut‐derived strains were isolated from stool samples of healthy people, and 80 clinical strains were isolated from sputum and ascites samples of patients. *E. coli* Top 10 chemically competent cells were purchased from GenStar. All strains were cultured in the Luria‐Bertani (LB) medium at 37°C with agitation at 200 rpm. Antibiotics were added at appropriate concentrations, including kanamycin (50 μg/ml) and gentamicin (20 μg/ml), as required.

### Phage isolation and purification

The *K. pneumoniae* phages were isolated from hospital wastewater or domestic sewage using the double‐layer agar method^40^, ^41^. Briefly, the LB medium was mixed with raw sewage (50%, vol/vol) and *K. pneumoniae* culture was added to the mixtures (1:20, vol/vol), followed by overnight incubation at 37°C. The supernatant obtained by centrifugation (10,000 rpm, 5 min) was then filtered through a 0.22 μm filter (Millipore Corp.). The filtrate was mixed with *K. pneumoniae* culture in molten 0.6% soft agar and plated on 1.2% bottom agar, incubated overnight at 37°C. Plaque formation was enumerated the following day. Single plaques were selected and purified through three rounds of plating. The purified single plaques were propagated in liquid culture (OD_600_ of 0.5) of *K. pneumoniae*. The supernatant was filtered to obtain phage stocks, which were stored at 4°C.

### Phage DNA extraction, sequencing, and annotation

High‐titer phage stocks (≥10^9^ PFU/ml) were used for DNA extraction using the Vazyme genomic DNA extraction kit. Sequencing was performed on the NovSeq 6000‐PE150 platform (Novogene). The phage genomes were assembled using Spades^42^ and annotated using Prokka^43^.

### Analysis of the phylogenetic relationship of phages and *K. pneumoniae* strains

To reveal the evolutionary relationships of phages, we constructed a Dice distance matrix‐based phylogenetic tree for the phages. In detail, we performed an all‐versus‐all comparison of all the phages based on their protein sequences by using Diamond (v.2.0.7)^44^ in a more sensitive mode. Then, we calculated a Dice distance matrix using the method as previously reported^45^. Subsequently, the Dice distance matrix was used to construct a neighbor‐joining tree using the ape package (v.5.0) of R^46^. As for the phylogenetic tree of *K. pneumonia*, we firstly constructed a core‐gene set based on the protein sequences of all the *K. pneumonia* strains by using Roary (v3.13.0)^47^. Then, the concatenated sequences of the core genes were conducted to build a maximum‐likelihood tree by using RAxML (v8.2.12)^48^.

### Phage host range assay (spot test)

The host range of phages was determined by the spot test^40^, ^41^. Briefly, bacterial strains were incubated to OD_600_ of 1.0, and 200 μl of culture plus soft agar was poured onto bottom agar plates. A quantity of 3 μl of phage stocks (10^9^ PFU/ml) was spotted onto plates for incubation at 37°C overnight. The lytic activity against each strain was determined by assessing the formation of visible plaques.

### TEM observation

The high‐purity phage supernatant was prepared for TEM analysis by staining with uranyl acetate. Images were collected using a JEM‐1400 transmission electron microscope (JEOL) under 80 kV conditions to observe the morphology of phages.

### Bacterial growth curves under the pressure of phage

To evaluate in vitro lytic ability of different phages or the phage cocktail against hvKp strains, the growth curves were determined using an automated growth curve analyzer (Bioscreen C). For this assay, 100 μl of OD_600_ = 0.2 bacterial culture (equivalent to 10^7^ CFU) was transferred to 100‐well flat plates (Honeycomb Microplate) and mixed with phage solution (single phage: 100 μl, double‐phage cocktail: 50 μl for each, triple‐phage cocktail: 33.3 μl for each), resulting in a final MOI of 1.0. Bacteria plus SM Buffer (Coolaber) without phages served as control. OD_600_ values were measured every hour, lasting for 24–72 h with shaking periodically. Experiments were conducted in triplicates, and inhibition curves were plotted with the average values of triplicates.

### Screening of phage‐resistant strains

To screen phage‐resistant strains, we used Kp067 as the parent strain. The strain was mixed with the phage at MOI of 10 and incubated until the bacterial culture became visibly turbid. The potential phage‐resistant populations were subsequently diluted and plated to screen out single colonies. These bacterial colonies against phages were further verified using the double‐layer agar plate method.

### Genome sequencing and mutation analysis for phage‐resistant strains

The genomic DNA of the phage‐resistant mutants (Kp067‐M1, Kp067‐M2, Kp067‐M3) was extracted using the TIANGEN genomic DNA purification kit. Subsequently, whole genome sequencing was carried out on the PromethION platform (NextOmics) and NovSeq 6000‐PE150 platform (Novogene) to obtain the Nanopore long reads and Illumina paired‐end reads. The complete genomes were firstly assembled based on the long reads by Flye^49^ and polished by the paired‐end reads through Nextpolish^50^. Then, the comparative genomic analysis was performed for the complete genomes of phage‐resistant isolates versus the genome of the wild type Kp067 (GenBank accession no. NZ_CP097651.1‐NZ_CP097653.1) through progressiveMauve^51^. The genomic variations were extracted by using an in‐house developed Perl script. Additionally, the mutations were validated by mapping the raw reads and calculating the read depth.

### Construction of gene knockout mutants and complemented strains

All gene knockout mutants were obtained using the CRISPR‐Cas9 combined with the λRed method^52^. Briefly, we constructed this knockout system including pCOLADRed (pCOLADuet‐1 plus λRed system) and pCas9gRNA (modified from pdCas9gRNA) plasmids, carrying their respective kanamycin and gentamycin resistance markers (Table S4). The pCOLADRed expresses the λRed recombination enzyme for improving the homologous recombination ability, and the pCas9gRNA expresses Cas9 and sgRNAs targeting the desired gene for eliminating the non‐recombinant wild‐type. The pCOLADRed was introduced into Kp067 by electroporation, simultaneously inducing λRed recombinase expression with a concentration of 1% arabinose. Then, the pCas9gRNA and the repair template were electroporated into the pCOLADRed‐containing cells and cultured on LB plates harboring 20 μg/ml gentamycin and 50 μg/ml kanamycin. The correct colonies were verified by PCR and sequencing. The primers used for the construction or validation of *wcaJ/gmhB/wbbY* single, double, and triple mutants are listed in Table S5.

For Kp067 complementation, an expression plasmid pCm derived from the pdCas9gRNA backbone (deleted dCas9 nuclease gene) was used with an aTc (anhydrotetracycline) inducible promoter (Table S4). The *wcaJ*, *gmhB*, and *wbbY* were separately introduced into pCm, obtaining the respective complemented plasmids pCm‐wcaJ/gmhB/wbbY through PCR and sequencing verification (Table S5). These plasmids were then transformed into the corresponding gene‐deficient mutants for complementation experiments.

### LPS extraction and electrophoresis

Kp067 and its gene knockout mutants were cultured overnight and LPS was extracted using an LPS extraction kit (iNtRON Biotechnology) according to the manufacturer's instructions. Subsequently, the samples were electrophoresed on 15% sodium dodecyl sulfate‐polyacrylamide gels electrophoresis (SDS‐PAGE) and visualized by silver staining (Solarbio).

### Preparation of bacterial culture and phage cocktails for mouse models

Overnight cultures of Kp067 were diluted in fresh LB (1:100) and cultivated at 37°C until reaching an OD_600_ of 1.0. The bacterial cultures were adjusted to 1 × 10^8^ CFU/ml in PBS. The phage stocks of RCIP0041, RCIP0002, and RCIP0070 were suspended in SM buffer to desired concentrations. The phage cocktail concentration of 1 × 10^8^ PFU/ml with RCIP0041, RCIP0002, and RCIP0070 was used at a volume ratio of 1:1:1.

### Construction of the mouse infection model and treatment with the phage cocktail

Male SPF BALB/c mice 6–8 weeks old (Charles River Corp.) were used for establishing murine models. Mice were housed in separate cages with a maximum of five mice. Then, mice were randomly grouped into non‐infected, infected, and treatment groups, with 6 mice per group^53^, ^54^, ^55^. Then, 100 μl of the 1 × 10^8^ CFU/ml Kp067 culture was orally inoculated into each mouse of the infected group and treatment group. While the control group was orally inoculated with 100 μl of PBS. One hour after infection, the treatment group was orally cured with 100 μl of the 1 × 10^8^ PFU/ml phage cocktail. Survival rates, weight percentages, and gut bacterial loads of the mice were monitored and recorded for a period of 7 days. To quantify gut bacterial loads, approximately two fresh stool pellets from each mouse were collected and suspended in 1 ml of sterile PBS. The samples were thoroughly mixed until no sediment was observed, followed by centrifugation to obtain the supernatant for bacteria counting. For counting, 10‐fold serial dilutions in PBS were performed, and 10 μl of the dilutions was plated onto the SCAI (Simmons Citrate Medium with 1% inositol) medium^56^ supplemented with ampicillin (25 μg/ml) and incubated overnight at 37°C for specific screening of *K. pneumoniae*.

### Statistical analysis

Statistical analyses in this study were performed with GraphPad Prism 7.0 (GraphPad Software, Inc.). *p* values were calculated by using a one‐way analysis of variance (ANOVA) and two‐tailed unpaired Student's *t* test. Data were considered significant when *p* values were below 0.05, as indicated.

## AUTHOR CONTRIBUTIONS

**Yongqi Mu**: Data curation (equal); methodology (equal); validation (equal); visualization (equal); writing—original draft (equal); writing—review and editing (equal). **Yuqin Song**: Formal analysis (equal); software (equal); supervision (equal); visualization (equal); writing—original draft (equal); writing—review and editing (equal). **Xueru Tian**: Data curation (equal); validation (equal); writing—review and editing (supporting). **Zixuan Ding**: Data curation (equal); validation (equal); writing—review and editing (supporting). **Shigang Yao**: Methodology (equal); resources (equal); writing—review and editing (supporting). **Yi Li**: Methodology (equal); validation (equal); writing—review and editing (supporting). **Chao Wang**: Methodology (supporting); validation (supporting); writing—review and editing (supporting). **Dawei Wei**: Methodology (supporting); validation (supporting); writing—review and editing (supporting). **Waldemar Vollmer**: Conceptualization (supporting); writing—review and editing (supporting). **Gang Zhang**: Conceptualization (equal); investigation (equal); methodology (equal); project administration (equal); supervision (equal); writing—original draft (equal); writing—review and editing (equal). **Jie Feng**: Conceptualization (equal); funding acquisition (equal); project administration (equal); writing—review and editing (equal).

## ETHICS STATEMENT

All animal studies and procedures have conformed to research ethics guidelines of the Institute of Microbiology, Chinese Academy of Sciences (IMCAS), China, on the use of animals in research. Mice were housed and bred in specific pathogen‐free (SPF) mouse facilities in compliance with research ethics guidelines. All experiments involved in animals have been approved by the Research Ethics Committee of IMCAS, NO. APIMCAS2022168.

## CONFLICT OF INTERESTS

The authors declare no conflict of interests.

## Supporting information

Supporting information.

Supporting information.

Supporting information.

Supporting information.

Supporting information.

Supporting information.

Supporting information.

Supporting information.

Supporting information.

Supporting information.

Supporting information.

Supporting information.

Supporting information.

Supporting information.

## Data Availability

Genomic sequence data for the phage‐resistant mutants of the *K. pneumonia* Kp067 strain used in this study have been deposited into the National Center for Biotechnology Information (NCBI; https://www.ncbi.nlm.nih.gov/) under the BioProject accession no. PRJNA1100799. The genomic data for the three phages used in this study were deposited into the GenBank with accession no. OR532835 (RCIP0041), OR532796 (RCIP0002), and OR532864 (RCIP0070).
