# Supplementary material for: Leveraging collateral sensitivity to counteract the evolution of bacteriophage resistance in bacteria
Source: mLife. 2025 Mar 18;4(2):143–54. doi: 10.1002/mlf2.70003 (PMC12042119; doi:10.1002/mlf2.70003)
Supplement: Supplementary file 1 — Supporting information. [file MLF2-4-143-s006.pdf]

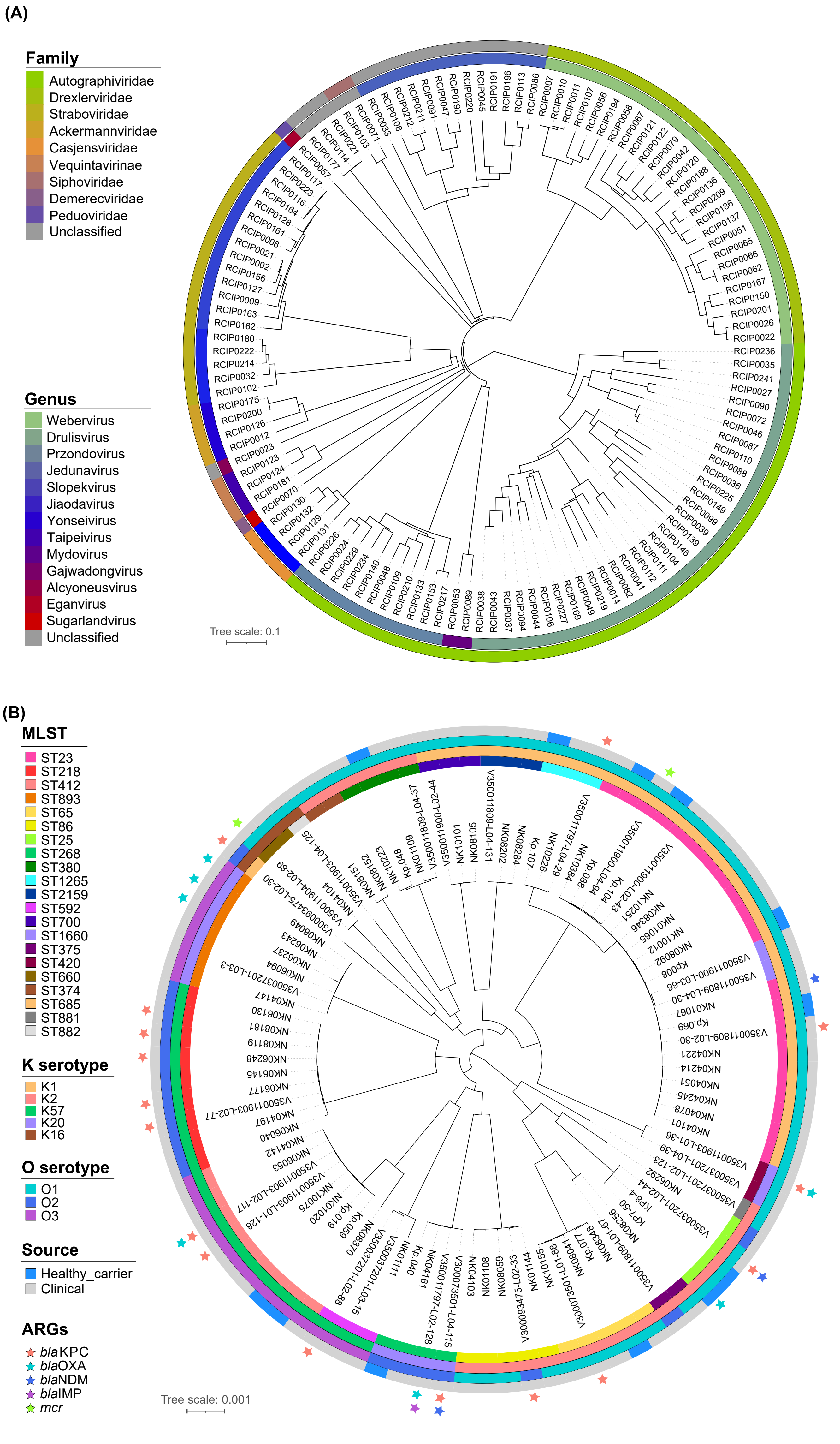
(A)
Family
Autographiviridae
Drexlerviridae
Straboviridae
Ackermannviridae
Casjensviridae
Vequintavirinae
Siphoviridae
Demerecviridae
Peduoviridae
Unclassified
Genus
Webervirus
Drulisvirus
Przondovirus
Jedunavirus
Slopekvirus
Jiaodavirus
Yonseivirus
Taipeivirus
Mydovirus
Gajwadongvirus
Alcyoneusvirus
Eganvirus
Sugarlandvirus
Unclassified
RCIP0191
RCIP0196
RCIP0113
RCIP0086
RCIP0007
RCIP0010
RCIP0011
RCIP0107
RCIP0056
RCIP0194
RCIP0058
RCIP0067
RCIP0121
RCIP0122
RCIP0079
RCIP0042
RCIP0120
RCIP0188
RCIP0136
RCIP0209
RCIP0186
RCIP0137
RCIP0051
RCIP0065
RCIP0066
RCIP0062
RCIP0167
RCIP0150
RCIP0201
RCIP0026
RCIP0022
RCIP0236
RCIP0035
RCIP0241
RCIP0027
RCIP0090
RCIP0072
RCIP0046
RCIP0087
RCIP0110
RCIP0088
RCIP0036
RCIP0225
RCIP0149
RCIP0099
RCIP0039
RCIP0139
RCIP0146
RCIP0104
RCIP0111
RCIP0112
RCIP0041
RCIP0082
RCIP0014
RCIP0219
RCIP0049
RCIP0169
RCIP0227
RCIP0106
RCIP0044
RCIP0094
RCIP0037
RCIP0043
RCIP0038
RCIP0089
RCIP0053
RCIP0217
RCIP0153
RCIP0133
RCIP0210
RCIP0109
RCIP0048
RCIP0140
RCIP0234
RCIP0229
RCIP0024
RCIP0226
RCIP0131
RCIP0129
RCIP0132
RCIP0130
RCIP0070
RCIP0181
RCIP0124
RCIP0123
RCIP0023
RCIP0012
RCIP0126
RCIP0200
RCIP0175
RCIP0102
RCIP0032
RCIP0214
RCIP0222
RCIP0180
RCIP0162
RCIP0163
RCIP0009
RCIP0127
RCIP0156
RCIP0002
RCIP0021
RCIP0008
RCIP0161
RCIP0128
RCIP0164
RCIP0116
RCIP0223
RCIP0117
RCIP0057
RCIP0177
RCIP0114
RCIP0221
RCIP0103
RCIP0071
RCIP0033
RCIP0108
RCIP0212
RCIP0211
RCIP0091
RCIP0047
RCIP0190
RCIP0220
RCIP0045
Tree scale: 0.1
(B)
MLST
ST23
ST218
ST412
ST893
ST65
ST86
ST25
ST268
ST380
ST1265
ST2159
ST592
ST700
ST1660
ST375
ST420
ST660
ST374
ST685
ST881
ST882
K serotype
K1
K2
K57
K20
K16
O serotype
O1
O2
O3
Source
Healthy_carrier
Clinical
ARGs
blaKPC
blaOXA
blaNDM
blaIMP
mcr
Tree scale: 0.001
