# Supplementary material for: Leveraging collateral sensitivity to counteract the evolution of bacteriophage resistance in bacteria
Source: mLife. 2025 Mar 18;4(2):143–54. doi: 10.1002/mlf2.70003 (PMC12042119; doi:10.1002/mlf2.70003)
Supplement: Supplementary file 2 — Supporting information. [file MLF2-4-143-s013.pdf]

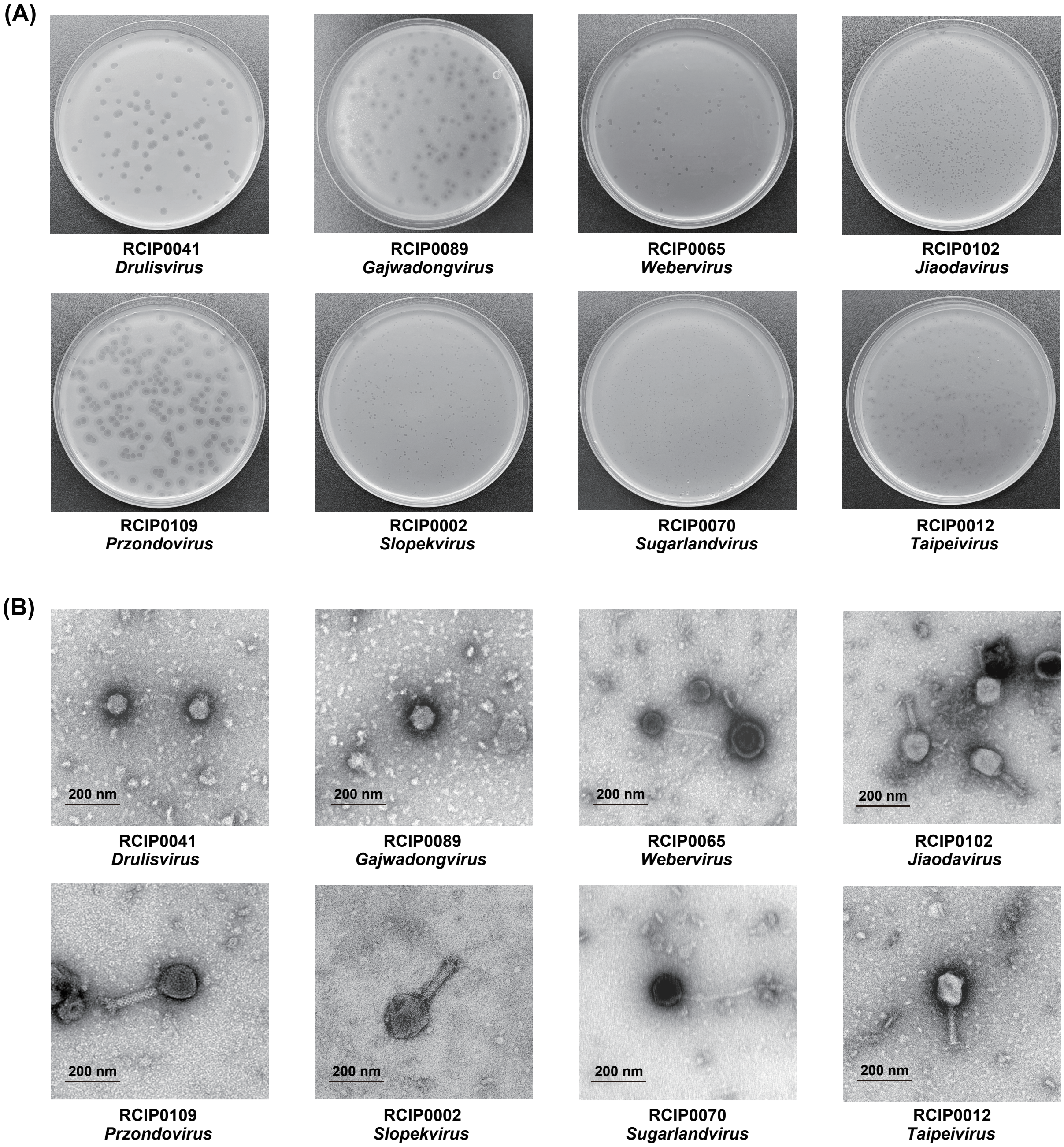
(A)
RCIP0041
Drulisvirus
RCIP0089
Gajwadongvirus
RCIP0065
Webervirus
RCIP0102
Jiaodavirus
RCIP0109
Przondovirus
RCIP0002
Slopekvirus
RCIP0070
Sugarlandvirus
RCIP0012
Taipeivirus
(B)
200 nm
RCIP0041
Drulisvirus
RCIP0089
Gajwadongvirus
RCIP0065
Webervirus
RCIP0102
Jiaodavirus
RCIP0109
Przondovirus
RCIP0002
Slopekvirus
RCIP0070
Sugarlandvirus
RCIP0012
Taipeivirus
