# Supplementary figures and images for: Leveraging collateral sensitivity to counteract the evolution of bacteriophage resistance in bacteria
Source: mLife. 2025 Mar 18;4(2):143–54. doi: 10.1002/mlf2.70003 (PMC12042119; doi:10.1002/mlf2.70003)

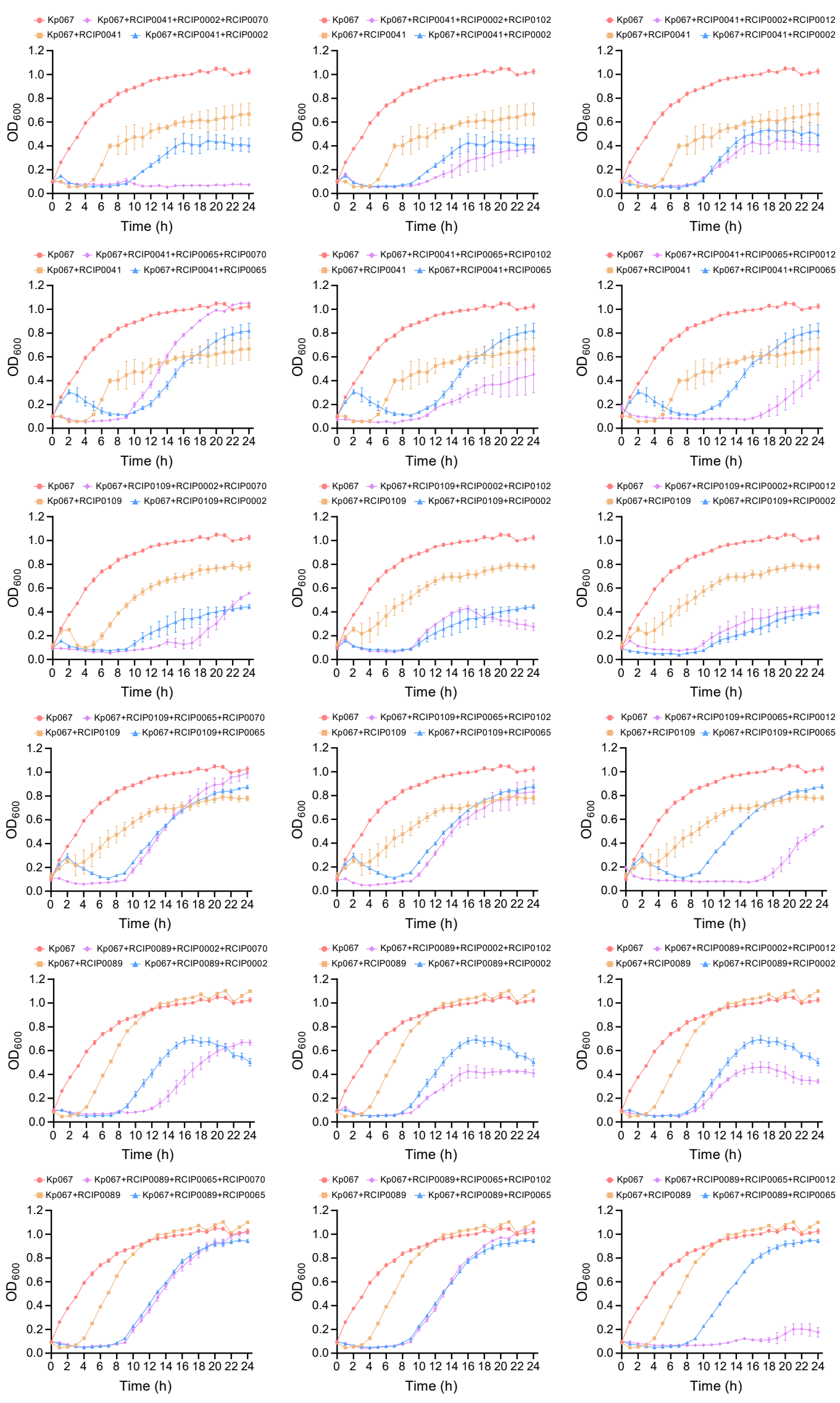

Supplement: Supplementary file 3 — Supporting information. [file MLF2-4-143-s001.pdf]

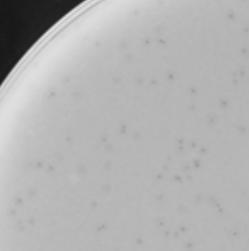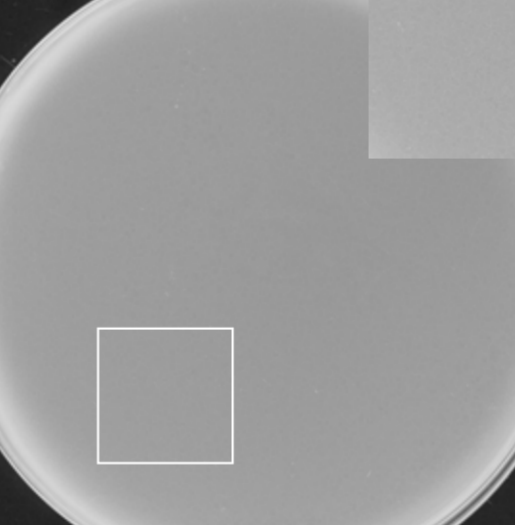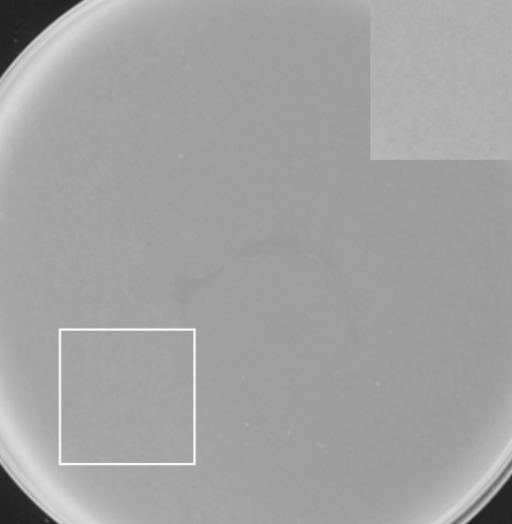

Supplement: Supplementary file 4 — Supporting information. [file MLF2-4-143-s007.pdf]

**(A)**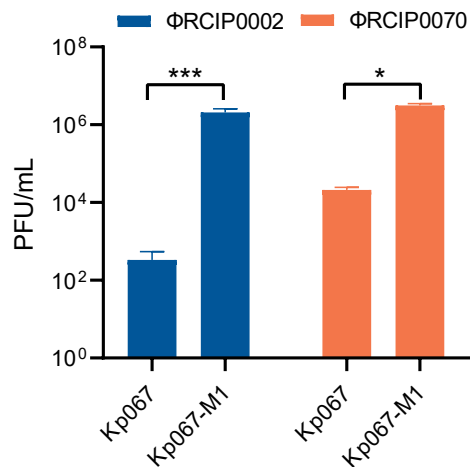**(B)**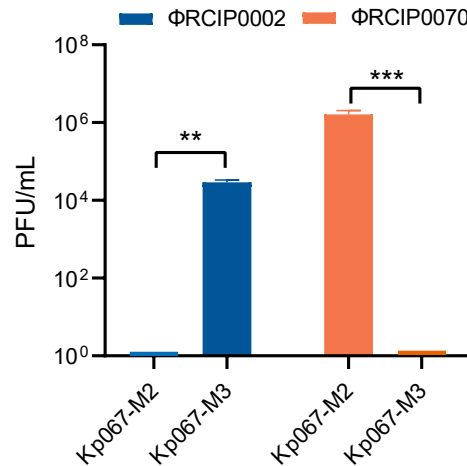**(C)**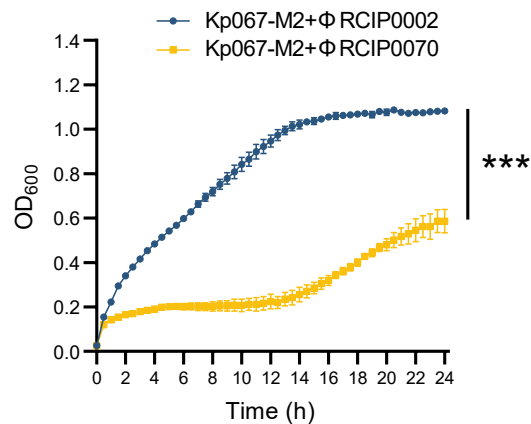**(D)**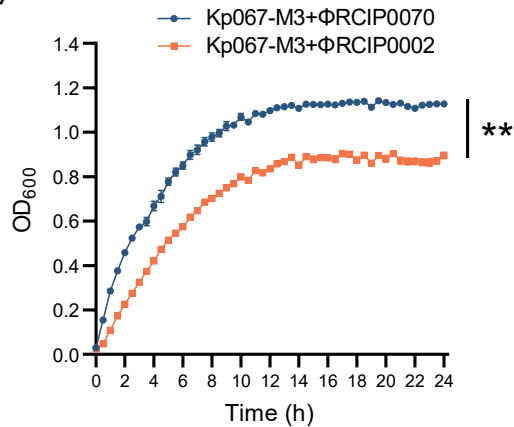

Supplement: Supplementary file 5 — Supporting information. [file MLF2-4-143-s008.pdf]

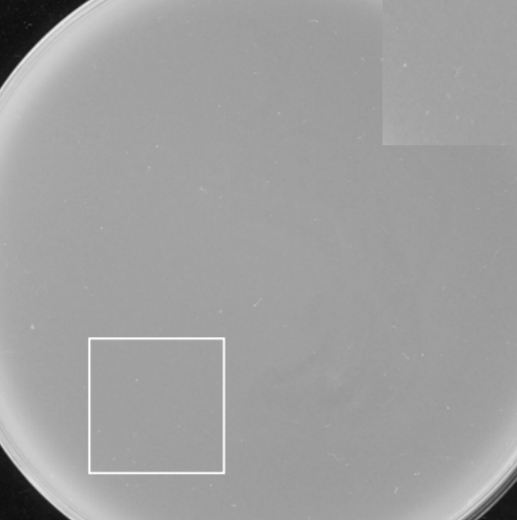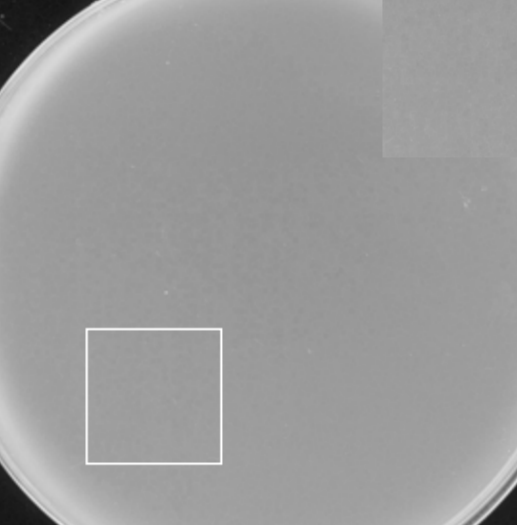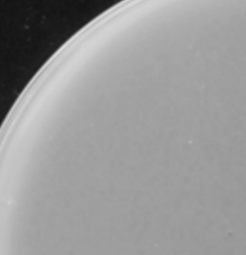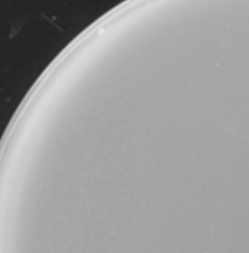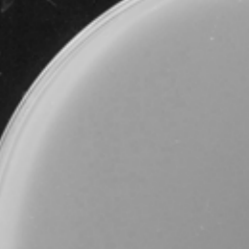

Supplement: Supplementary file 6 — Supporting information. [file MLF2-4-143-s011.pdf]

(A)

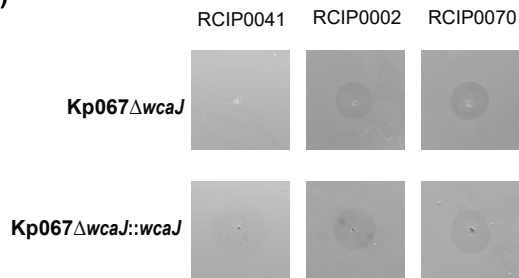

(B)

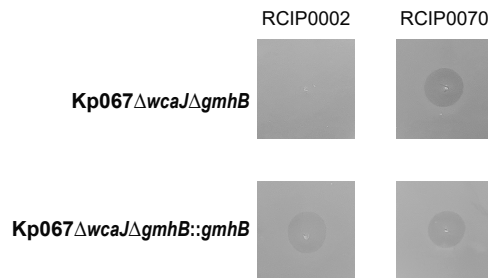

(C)

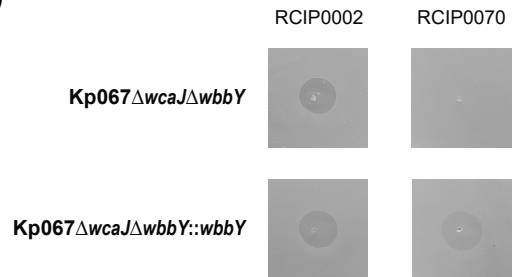

(D)

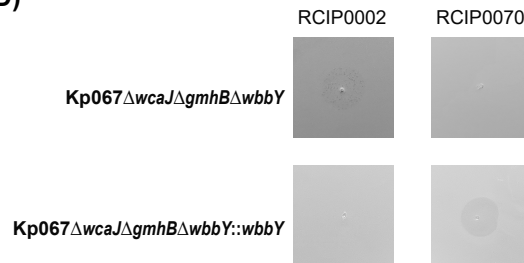

Supplement: Supplementary file 7 — Supporting information. [file MLF2-4-143-s003.pdf]

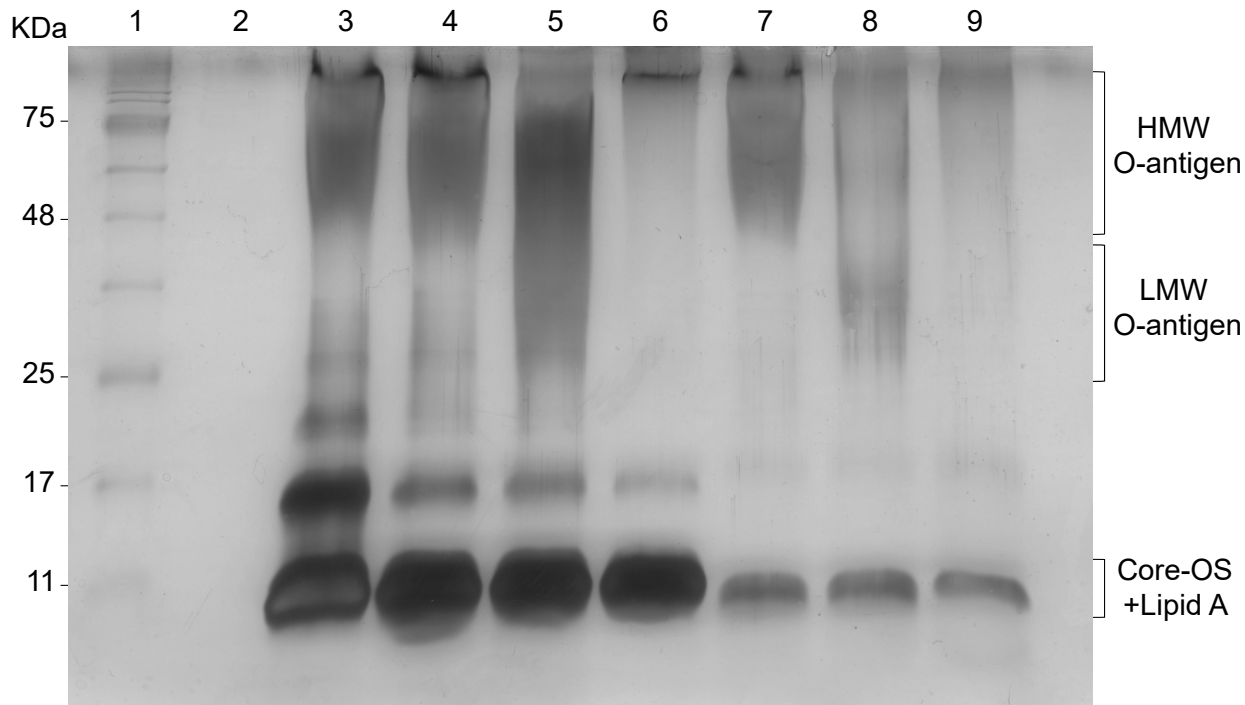

Supplement: Supplementary file 8 — Supporting information. [file MLF2-4-143-s014.pdf]
