# Supplementary material for: Leveraging collateral sensitivity to counteract the evolution of bacteriophage resistance in bacteria
Source: mLife. 2025 Mar 18;4(2):143–54. doi: 10.1002/mlf2.70003 (PMC12042119; doi:10.1002/mlf2.70003)
Supplement: Supplementary file 14 — Supporting information. [file MLF2-4-143-s002.docx]

**Legends of supplementary materials**

**Figure S1. Phylogenetic trees of phages and hvKp isolates.** **(A)** The Neighbor-Joining tree of 124 phages based on the Dice distance matrix. The color strip from the inside out represented genus and family of all the phages. **(B)** Phylogenic tree of 90 hvKp strains based on core-genes and clustered by midpoint rooting. Rings from inside to outside represented MLST types, K serotypes, O serotypes, isolation sources and the carriage of antibiotic-resistant genes of carbapenem and colistin, respectively.

**Figure S2.** Plaque morphology **(A)** and electron micrographs **(B)** of the eight selected phages: ΦRCIP0041, ΦRCIP0109, ΦRCIP0089, ΦRCIP0002, ΦRCIP0065, ΦRCIP0070, ΦRCIP0102 and ΦRCIP0012. The taxonomy information at genus level were labeled for each phage.

**Figure S3. Growth curves of uninfected and phage-infected Kp067 cultures.** The x-axis denoted the detection time at 24▒hours, while the y-axis measured the optical density (OD_600_). The displayed data represented the means and standard deviations (SD) from three separate experiments.

**Figure S4. The lytic ability of phages ΦRCIP0041, ΦRCIP0002 and ΦRCIP0070 to Kp067 and its mutants.** Each row represented Kp067 and its mutants, while each column represented the phages ΦRCIP0041, ΦRCIP0002, and ΦRCIP0070. The upper panel displayed lytic ability detected by the double-layer agar plate method, and the lower panel showed a spot test with a 10^-1^-10^-5^ -fold dilution of phages.

**Figure S5. Assessment of phage-induced lysis in Kp067 and its mutants.** **(A)** The infection efficiency of phages ΦRCIP0002 and ΦRCIP0070 in Kp067 and its mutant, Kp067-M1. **(B)** The infection efficiency of phages ΦRCIP0002 and ΦRCIP0070 in Kp067-M2 and Kp067-M3. **(C-D)** Growth curves of phage ΦRCIP0002 and ΦRCIP0070 infected Kp067-M2 and Kp067-M3 cultures. Error bars represent SD. *: *p*▒<▒0.05; **: *p*▒<▒0.01; ***: *p*▒<▒0.001.

**Figure S6. The lytic ability of phages ΦRCIP0041, ΦRCIP0002 and ΦRCIP0070 to Kp067 and its gene knockout mutants.** Each row represented Kp067 and its gene knockout mutants, while each column represented the phages ΦRCIP0041, ΦRCIP0002 and ΦRCIP0070. The upper panel displayed lytic ability detected by the double-layer agar plate method, and the lower panel showed a spot test with a 10^-1^-10^-5^ -fold dilution of phages.

**Figure S7. Complementation of gene-deficient mutants.** **(A)** Complementing the *wcaJ* gene in Kp067∆*wcaJ* strain. **(B)** Complementing the *gmhB* gene in Kp067∆*wcaJ*∆*gmhB* strain. **(C)** Complementing the *wbbY* gene in Kp067∆*wcaJ*∆*wbbY* strain. **(D)** Complementing the *wbbY* gene in Kp067∆*wcaJ*∆*gmhB*∆*wbbY* strain. Visible plaques represented sensitivity and no plaque represented resistance.

**Figure S8. Silver-stained SDS-PAGE profiles for LPS of Kp067 and its gene knockout mutants.** Lane 1 to lane 9 represented maker, blank, Kp067, Kp067∆*wcaJ*, Kp067∆*wcaJ*∆*wbbY*, Kp067∆*wcaJ*∆*wbbN*, Kp067∆*wcaJ*∆*gmhB*, Kp067∆*wcaJ*∆*gmhB*∆*wbbY*, Kp067∆*wcaJ*∆*gmhB*∆*wbbY*∆*wbbN*, respectively. HMW, high molecular weight; LMW, low molecular weight; Core-OS, core-oligosaccharide.

**Table S1**. The information of 90▒*K. pneumoniae* strains used in this study.

**Table S2**. The detailed information of 124 phages used in this study.

**Table S3**. The distribution of different O serotypes in 1760 public available *K. pneumoniae* strains.

**Table S4**. Strains and plasmids used in the work.

**Table S5**. The primers of gene knockout and complementation.
